# Sex-Specific Transcriptomic Profiles in Psoriatic Lesions: A Large-Scale Integrative Study

**DOI:** 10.3390/ijms27104439

**Published:** 2026-05-15

**Authors:** Edia Stemmer, Liat Anabel Sinberger, Tair Lax, Guy Shrem, Inbal Mor, Mali Salmon-Divon

**Affiliations:** 1 Department of Molecular Biology, Ariel University, Ariel 40700, Israel; stemmere@ariel.ac.il (E.S.); inbalmor@ariel.ac.il (I.M.); 2 Fertility Clinic, North Distinct, Clalit Health Services, Migdal Ha’emek 2303000, Israel; 3 The Azrieli Faculty of Medicine, Bar Ilan University, Safed 1311502, Israel; 4 Adelson School of Medicine, Ariel University, Ariel 40700, Israel

**Keywords:** inflammation, biomarkers, psoriasis, pathogenesis, sex-specific expression, IL-17 signaling

## Abstract

Psoriasis, a chronic inflammatory skin disease affecting men and women equally, presents distinct gender-based differences in severity and treatment response. While molecular mechanisms underlying psoriasis are well-studied, sex-specific differences remain largely unexplored. To address this, we conducted a comprehensive analysis of transcriptomic data from lesional psoriasis skin and healthy controls, comparing male and female cohorts. Our findings reveal 2760 overlapping differentially expressed genes (DEGs) between sexes, highlighting shared pathways like IL-17 signaling and Th17 differentiation. However, sex-specific pathways emerged, including male-enriched PI3K-Akt signaling and chemokine receptor activity, and female-enriched glycolysis and AHR-NRF2 pathways. Upstream regulator analysis identified sex-specific drivers, including VEGFA activation and CFTR inhibition in males, and AHR activation and FGF21 inhibition in females. Notably, Regulatory T cells (Tregs) and neutrophil abundance differed by sex, aligning with disease severity trends. These results highlight sex-associated molecular and cellular disparities that may be relevant to understanding differences in disease manifestation and treatment response. As an exploratory, hypothesis-generating transcriptomic analysis, this study lays the groundwork for future experimental and clinical validation of sex-specific mechanisms in psoriasis.

## 1. Introduction

Psoriasis is a chronic inflammatory skin disease affecting both men and women equally that can develop at any age [1]. It occurs worldwide and imposes a significant burden on individuals and society, particularly in high-income and high-Socio-Demographic-Index (SDI) countries in North America and Europe [2].

Psoriasis manifests in various clinical phenotypes, with chronic plaque psoriasis (psoriasis vulgaris) being the most common and easily recognizable [3]. Psoriatic lesions typically appear as well-demarcated, erythematous scaly patches or thick, raised plaques with silvery scales [4]. These features result from increased keratinocyte proliferation, abnormal differentiation, and incomplete cornification, leading to the retention of nuclei in the stratum corneum (parakeratosis). The lesions are also characterized by an inflammatory infiltrate of dendritic cells, macrophages, and T cells in the dermis, with neutrophils and some T cells in the epidermis, and increased vascularity with tortuous capillaries contributing to the redness [5].

Despite extensive research highlighting the involvement of genetic and environmental factors—such as stress, microorganisms, drugs, trauma, and smoking—in the etiology and pathogenesis of psoriasis, the exact mechanisms remain unclear [6]. However, several key immunopathologic pathways are known to be involved, including extracellular signaling pathways like TNF, IL-17, and IL-23, as well as intracellular pathways such as NF-κB, JAK-STAT and TYK2 [7].

The therapeutic approach to psoriasis varies substantially, ranging from topical agents for mild cases to systemic treatments for moderate disease and advanced biologics for severe cases targeting cytokines such as TNF-α [8].

Sex-specific manifestations of psoriasis have gained increasing attention in clinical research. While the overall prevalence is comparable between sexes, significant differences exist in disease expression and severity patterns [9]. Male patients consistently demonstrate higher psoriasis area and severity index (PASI) scores [10] and require more intensive therapeutic interventions, particularly biologics [11]. Recent investigations have also uncovered sex-specific variations in psoriatic arthritis presentation and progression, suggesting the need for sex-specific therapeutic approaches [12].

Sex differences in terms of hormones and gene expression are considered to be the main reasons for differences in diseases [13,14]. Additionally, variability in drug metabolism, along with social and cultural factors related to gender differences, may also have a major impact on the development and experience of psoriasis.

Despite the growing recognition that biological sex is a major determinant of immune function, drug metabolism, and disease trajectory [13,14], sex-specific molecular mechanisms in psoriasis remain poorly characterized at the transcriptomic level. Most transcriptomic studies of psoriasis have not stratified analyses by sex, or have been underpowered to detect sex-specific effects due to limited individual sample sizes. While clinical data consistently document greater disease severity in males [9,10,11], and a recent comprehensive review has catalogued sex differences in biomarkers and biologic mechanisms in psoriatic diseases [15], the underlying transcriptomic landscape driving these sex differences has not been systematically investigated. The main challenge in addressing this gap is the need for large, sex-stratified sample sets, which are difficult to achieve within single-study designs.

To address this gap, we performed a large-scale integration of uniformly reprocessed raw transcriptomic data from lesional skin biopsies of psoriasis patients and healthy controls, combining 13 independently conducted publicly available studies encompassing approximately 350 skin biopsies. This approach enabled sufficient statistical power to detect sex-associated gene expression patterns that would not be detectable in individual studies. To our knowledge, this is the first study to systematically investigate the transcriptomic landscape of sex differences in psoriasis using publicly available bulk RNA-seq data at this scale.

## 2. Results

**Figure 1:**
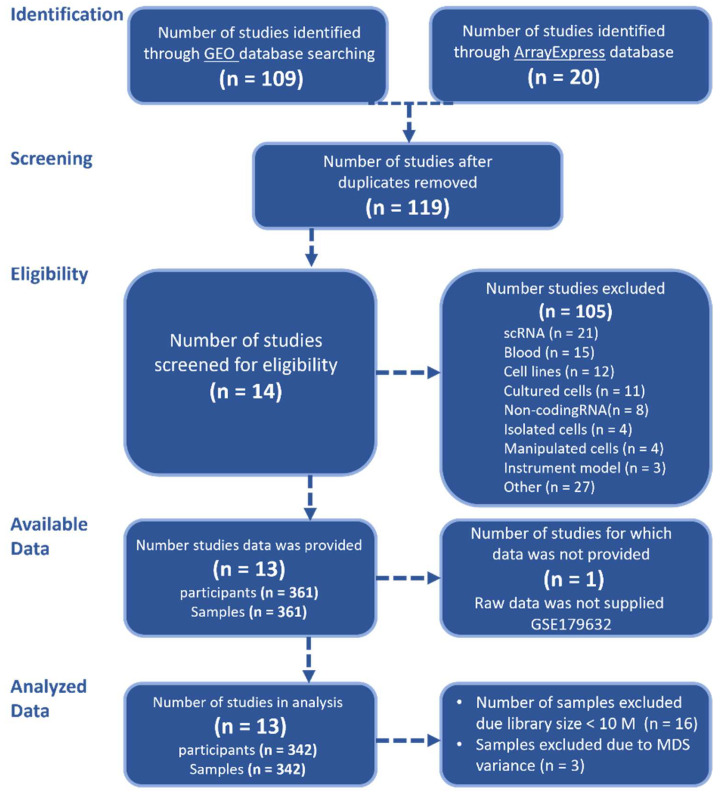
PRISMA flow diagram. PRISMA: Preferred Reporting Items for Systematic Reviews and Meta-Analyses.

Following the PRISMA recommendations in the GEO and ArrayExpress databases, our search identified 13 studies containing transcriptomic data of 342 skin samples that met our inclusion criteria (Figure 1). Only four studies provided detailed information regarding sex. For the remaining nine studies (marked with an asterisk in Table 1), we used a logistic regression model based on gene expression of specific markers to infer sample sex. Multidimensional Scaling (MDS) plots before and after the inference are depicted in Supplementary Figure S1. Table 1 provides a detailed summary of the distribution of psoriasis and control samples by sex for each study.

### 2.1. MDS Analysis Reveals Disease and Sex-Driven Expression Variation

**Figure 2:**
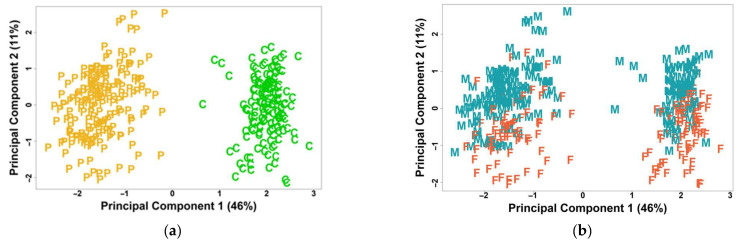
MDS analysis of normalized biopsy expression data, with batch effects removed. (**a**) Disease status—Psoriasis (P, yellow) vs. Control (C, green), and (**b**) Sex status—Female (F, orange) vs. Male (M, turquoise).

MDS analysis of biopsy expression levels was conducted after the removal of the study batch effect, revealing that the primary factor driving variation was the disease status, distinguishing between lesional psoriasis biopsies and control skin biopsies (Figure 2a). Subsequently, within each group, the next factor influencing variation was the sex status of the biopsies (Figure 2b).

Together, these findings establish that while psoriasis-associated transcriptomic changes are largely consistent between sexes, biological sex represents an additional and independent driver of expression variation within both the disease and control groups.

### 2.2. Pathway Enrichment and Upstream Regulator Analysis of DEGs

**Figure 3:**
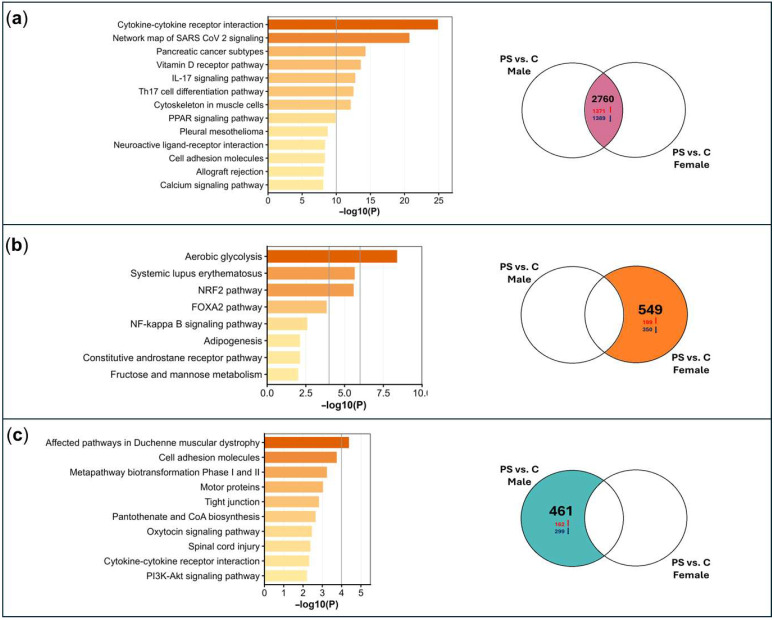
Metascape and Venn diagram of psoriasis DEGs. Metascape analysis is provided for each section of the Venn diagram, detailing the enrichment of biological terms and pathways specific to the gene sets. The Venn diagram shows DEGs between psoriasis (PS) and control (C) for both males and females, including overlaps. Arrows indicate DEG direction: red for upregulated and blue for downregulated. (**a**) Pathways for overlapping DEGs. (**b**) Pathways for DEGs unique to female psoriasis (not in males). (**c**) Pathways for DEGs unique to male psoriasis (not in females).

DEG analysis revealed 3309 DEGs (full list of DEGs is available upon request) in females with psoriasis compared to female controls, and 3221 DEGs in males with psoriasis compared to male controls. Of these, 2760 DEGs were common to both comparisons, with consistent directionality (upregulated or downregulated in both). Notably, the S100A family and IL17 genes were upregulated in both male and female psoriasis groups. Additionally, 549 DEGs were unique to females, while 461 DEGs were unique to males. The directional distribution of DEGs is illustrated in Figure 3a–c, showing the Venn diagrams sections of the two comparisons. Metascape analysis was conducted for each section of the Venn diagrams, examining pathways associated with the overlapping DEGs in male and female samples and those unique to each sex-specific comparison (psoriasis male vs. control male and psoriasis female vs. control female). For the overlapping DEGs, key pathways identified included the “Th17 cell differentiation pathway”, “IL-17 signaling pathway”, and “Cytokine-cytokine receptor interaction”, as well as pathways related to calcium regulation such as the “Calcium signaling pathway” and “Vitamin D receptor pathway” (Figure 3a). DEGs unique to psoriasis females were associated with sugar metabolism pathways, including “aerobic glycolysis” and “fructose and mannose metabolism”, as well as oxidative stress pathways like “NRF2” and “FOXA2”. “NF-kappa B signaling”, crucial for inflammation and immune activation, was also implicated (Figure 3b). For the male psoriasis comparison, distinct DEGs were found in pathways like “Cell adhesion molecules”, “Cytokine-cytokine receptor interaction”, “Tight junction” and “PI3K-Akt signaling pathway”, emphasizing the roles of inflammation, barrier function disruption, and cell proliferation in driving the pathology of psoriasis in males (Figure 3c).

To ensure that the observed findings were not driven by the imbalance between male and female participants, we conducted a sensitivity analysis using a balanced dataset, which produced consistent results.

Ingenuity Pathway Analysis (IPA) upstream regulator analysis, using an absolute Z score of 2 or greater and a *p*-value less than 0.05, identified regulators across various molecular types. Table 2 highlights regulators of molecule types related to immune responses (cytokines, transmembrane receptors), proliferation and differentiation (growth factors), and other metabolic and intracellular signaling pathways relevant to psoriasis pathogenesis that were found exclusively in one sex. It is worth noting that for all the regulators that were common to both sexes, the directionality of the effect was the same in the two groups; if a regulator was activated in males, it was also activated in females, and the same applied to inhibited regulators.

Overall, while core psoriasis pathways are shared between sexes, distinct DEG sets and upstream regulators define sex-specific transcriptomic programs that may contribute to differences in disease manifestation and severity.

### 2.3. Sex-Specific DEGs

Ten DEGs were identified in the interaction analysis between male and female psoriasis patients, as shown in Table 3. Among these, *SYT6*, *PSG4*, *NELL2*, *GPR15*, *MAP1LC3C* and *C10orf67* are protein-coding genes; *CYP4Z2P*, *RN7SL5P* and *CLCA3P* are pseudogenes; and *LINC01698* is a long non-coding RNA (lncRNA). Functional interpretations of the pseudogenes (*CYP4Z2P*, *RN7SL5P*, *CLCA3P*) and *C10orf67*—a gene with no currently annotated function—should be treated with caution, as experimental evidence supporting their roles in psoriasis or sex-specific biology remains limited. The enrichment of multiple pseudogenes and a lncRNA among the interaction genes may reflect regulatory roles that are not yet fully characterized. None of these genes are located on the sex chromosomes. Table 3 provides detailed information about these genes, obtained from the GeneCards database (www.genecards.org accessed on 24 November 2024) [16], demonstrating a high representation of proteins that are relevant to calcium signaling or intracellular signaling. The expression of these interaction genes is presented in boxplots in Supplementary Figure S2. GSEA of genes ranked based on their interaction fold change identified similar pathways that were detected as differentially enriched in psoriasis males as mentioned above. For example, bioenergetic pathways such as “Cell Adhesion Molecules,” “Glycolysis/Gluconeogenesis,” and “Oxidative Phosphorylation” were less enriched in male psoriasis, while proinflammatory pathways like “IL-17 Signaling” and “Chemokine Receptors Bind Chemokines” were more enriched. These pathway analyses are illustrated in Supplementary Figure S3.

In summary, the interaction analysis identified a small but biologically informative set of genes whose response to psoriasis differs between sexes, encompassing multiple gene biotypes and pointing to calcium signaling and immune regulation as candidate sex-differentiating processes.

### 2.4. Differential Cell Type Enrichment Between Sexes in Psoriasis

**Figure 4:**
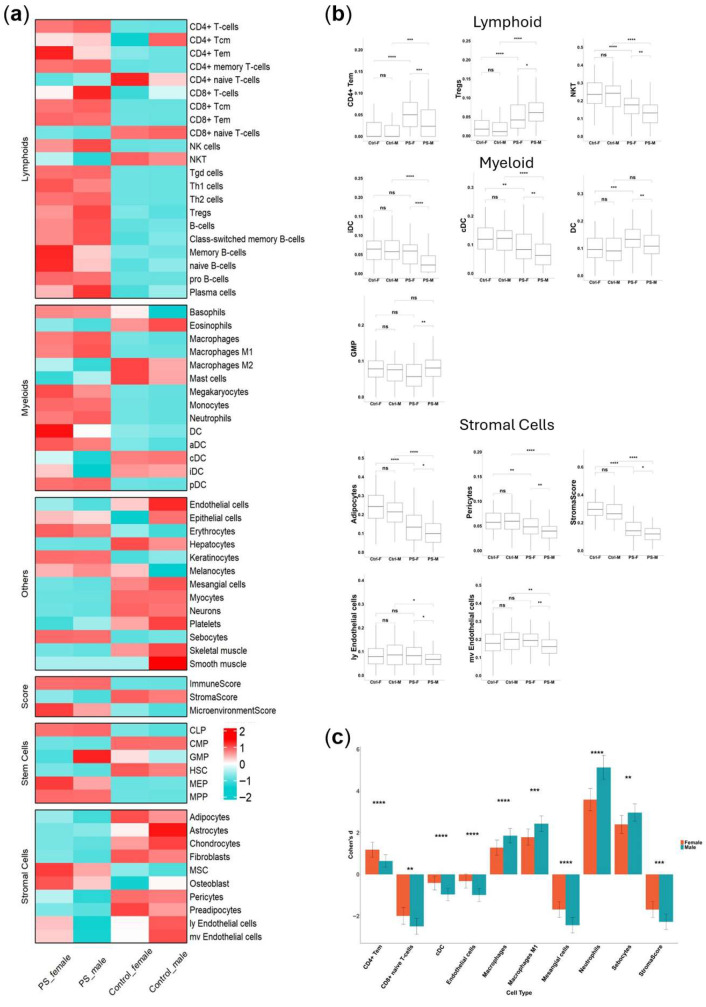
Cell-type enrichment analysis. (**a**) Heatmap of cell-type enrichment analysis showing the average xCell scores for each group: PS-Female, PS-Male, Control-Female, and Control-Male. (**b**) Boxplots illustrating significant sex-specific differences in psoriasis. Groups compared include PS-Female (PS-F), PS-Male (PS-M), Control-Female (Ctrl-F), and Control-Male (Ctrl-M). Statistical significance was assessed using Dunn’s test, with *p*-values adjusted using FDR correction. Asterisks indicate significance levels. (**c**) Barplots displaying Cohen’s D scores for cell types, representing the effect size of differences between groups. Cohen’s D was calculated separately for females (PS-F vs. Ctrl-F) and males (PS-M vs. Ctrl-M). For each comparison, the variances of Cohen’s D were estimated, and a z-statistic was computed to evaluate the differences between male and female effect sizes. *p*-values were derived from the z-statistics and corrected using FDR. The *y*-axis shows Cohen’s D values, while the *x*-axis lists cell types. Error bars represent confidence intervals. Significance levels: * *p* < 0.05, ** *p* < 0.01, *** *p* < 0.001, **** *p* < 0.0001, with “ns” for non-significant.

To explore possible sex-based variation in cell populations within the samples, we used the xCell webtool to perform cell-type enrichment analysis on bulk gene expression data, normalized for gene length, across four groups: psoriasis males, psoriasis females, control males, and control females. The heat map displayed in Figure 4a shows the average scores for each cell type across these groups. Psoriasis was associated with significant changes in various cell populations compared to the control, with increased immune and microenvironment scores and decreased stromal scores. While the overall patterns in the heatmap appear similar between sexes in both the psoriasis and control groups, a more detailed analysis reveals significant sex-based variations within the psoriasis group, as illustrated in Figure 4b.

Tregswere significantly higher in males compared to females, while NKT cells and CD4+ Tem cells were lower in males. Among myeloid cells, conventional dendritic cells (cDCs), immature dendritic cells (iDCs), and overall dendritic cells (DCs) were reduced in males, whereas granulocyte–monocyte progenitors (GMPs) were increased. Regarding stromal cells, most are significantly reduced in male psoriasis compared to females (Figure 4b).

Next, we sought to determine whether there are significant sex-based differences in microenvironment responses to psoriasis and how large those differences are. For this aim, we used Cohen’s D calculation, assessing how much the xCell enrichment scores differ between the psoriasis and control groups for each sex. The results of this analysis are shown in Figure 4c.

We observed elevated scores for neutrophils and M1 macrophages in male psoriasis, alongside reduced scores for cDCs. Mesangial cells significantly decreased in males, while sebocytes increased. CD4+ Tem cells were the only cell type that significantly increased in female psoriasis.

These results highlight the complex and sex-specific cellular alterations in psoriasis, underscoring the importance of considering sex when analyzing immune and stromal cell populations in this disease.

Collectively, these results indicate sex-specific differences in the immune and stromal cell composition of psoriatic lesions, with males showing patterns of elevated neutrophils, M1 macrophages, and Tregs that are broadly consistent with a more inflammatory and potentially more severe disease microenvironment.

## 3. Discussion

The present study reports a large-scale exploratory analysis of sex-associated transcriptomic differences in psoriatic lesions. As an observational meta-analysis of publicly available RNA-seq data, it is designed to generate hypotheses rather than to establish causal mechanisms, and all findings should be interpreted accordingly. Psoriasis is a chronic inflammatory skin disease affecting both men and women. Although many studies have examined the molecular mechanisms involved in psoriasis by transcriptomic analyses of healthy and psoriatic skin, sex-based differences remain largely unexplored, despite evidence suggesting that males often experience more severe disease [9]. To uncover the role sex plays in psoriasis, we compared gene expression between lesional psoriatic skin and healthy control skin from both males and females, using uniformly reprocessed samples from 13 different studies for a comprehensive analysis.

MDS analysis showed sex was second only to disease status as the main driver of gene expression differences, emphasizing its role in shaping disease outcomes and treatment responses.

The differential gene expression analysis in psoriasis highlights significant similarities and sex-specific differences in the underlying mechanisms of the disease. The identification of 2760 overlapping DEGs between male and female psoriasis patients suggests the involvement of a shared set of genes in the pathogenesis of psoriasis, pointing to common core pathways. These DEGs are notably linked to psoriasis-related pathways, such as Th17 cell differentiation, IL-17 signaling, and cytokine-cytokine receptor interactions, which are central to the inflammatory processes driving psoriasis. Furthermore, the involvement of vitamin D receptor and calcium signaling pathways also aligns with the importance of calcium metabolism in maintaining keratinocyte function [17,18]. Disrupted calcium gradients in the epidermis, often seen in psoriasis, are critical in impairing keratinocyte differentiation and skin barrier function [19]. Moreover, the enriched pathway “Chemokine receptors bind chemokines” identified in the male group is associated with intracellular calcium fluxes, which regulate chemotaxis and inflammatory responses [20]. Other pathways identified specifically in the male group related to tight junctions and cell adhesion molecules suggesting alterations in keratinocyte barrier integrity, which may also be tied to abnormal calcium regulation. In contrast, female-specific DEGs were enriched for bioenergetics pathways, including glycolysis and fructose metabolism. While these pathways have been associated with calcium transients and proliferation in other cellular contexts [21], we note that a direct link between glycolysis and calcium signaling in psoriatic keratinocytes has not been established in the present study. The enrichment of these pathways may potentially reflect a metabolic adaptation in female psoriasis, a hypothesis that requires targeted experimental validation. Indeed, increased expression of glycolysis-related genes is associated with disease severity modulation [22].

In the context of metabolic changes, we found the PI3K-Akt signaling pathway to be differentially enriched in the male group. This pathway has been reported to be activated in psoriatic keratinocytes, where elevated PI3K expression may contribute to Akt hyperactivity and potentially promote keratinocytes proliferation [23]. However, a direct causal link between the PI3K-Akt pathway and keratinocyte behavior cannot be established from transcriptomic data alone. The differential enrichment of this pathway in males is consistent with a potentially more proliferative disease phenotype, though this interpretation remains associative and requires experimental confirmation. Furthermore, in osteoblasts, calcium transients have been shown to activate glycolysis via AKT-related signaling [21], and a similar mechanism may conceivably operate in psoriatic skin. However, this remains speculative in the absence of targeted experimental evidence. Taken together, we propose that sex-specific regulation of metabolic and calcium-associated pathways may potentially contribute to differences in psoriasis pathogenesis between males and females, and we highlight these as hypotheses for future investigation.

The upstream regulator analysis revealed regulators specific to either males or females. A recent study on ion channels in psoriasis pathophysiology emphasizes CFTR’s role in epidermal cells, particularly in regulating fluid balance, reducing inflammation, and promoting keratinocyte differentiation [24,25]. In our study, CFTR was identified as an inhibited upstream regulator in male psoriasis, consistent with the possibility of a more severe disease course in males, though a direct causal relationship cannot be inferred from transcriptomic data alone. Activated transcription factors in males included TBX21 and STAT5A, which drive Th1 immune responses and cellular proliferation [26,27]. Growth factors such as VEGFA were also upregulated in our analysis. The inferred activation of VEGFA in male psoriasis is consistent with prior reports linking VEGFA to disease severity [28,29,30], and may point to more pronounced angiogenic activity in males; however, this association is observational and cannot be interpreted causally in the current study. In contrast, female-specific regulators included the activation of potassium ion channel KCNJ10, which plays a role in epithelial repair, particularly in cell migration and proliferation [31]. Furthermore, female psoriasis is marked by the activation of ligand-dependent nuclear receptors like AHR along with the inhibition of growth factors such as FGF21, which are involved in metabolic regulation and oxidative stress responses. The NRF2 pathway was enriched in our female-specific analysis. The AHR-NRF2 axis is likely anti-inflammatory, reducing the production of proinflammatory cytokines. AHR agonists, such as tapinarof, are currently used as therapeutic agents in psoriasis [32]. FGF21, a key regulator of keratinocyte migration and differentiation during wound healing [33], is typically elevated in the serum of psoriasis patients, correlating with disease severity [34]. However, in our data, FGF21 was identified as an inhibited upstream regulator in female psoriasis. The inhibited status identified here in females may potentially be consistent with a less severe disease phenotype, though this interpretation requires clinical validation. In summary, sex-specific upstream regulators highlight distinct molecular drivers of psoriasis in males and females, suggesting that therapeutic approaches should be tailored to these differences.

We identified 10 genes that showed an interaction between sex and disease, indicating that psoriasis influences these genes differently in males and females. Of these, six are known to be associated with psoriasis. *SYT6*, located in the *PTPN22* region, is linked to early-onset psoriasis [35] and is differentially methylated in psoriatic lesions [36]. *NELL2*, a calcium binding glycoprotein, is deregulated in psoriatic substitutes and found to be increased in atopic dermatitis (AD) compared to psoriasis. Upregulated by Th2 cytokines [37], *NELL2* prevents ER stress-induced cell death and promotes cell survival [38] by activating the ERK1/2 pathway, which is overactive in psoriasis lesions [39]. This activation supports cell survival and proliferation [40]. Notably, *NELL2* is upregulated in female psoriasis, suggesting a female-specific pathogenesis. *PSG4* was found to be highly expressed in male psoriasis in our data and is associated with generalized pustular psoriasis (GPP), a more severe form of psoriasis characterized by neutrophil/monocyte dominance and extensive systemic manifestations [41]. *GPR15* and its ligand, *GPR15LG*, are involved in skin lymphocyte homing and keratinocyte proliferation, with *GPR15LG* significantly elevated in psoriatic lesions [42,43,44]. *MAP1LC3C* showed potential links to autophagy, being downregulated in psoriatic lesions compared to normal skin [45]. *CYP4Z2P*, a pseudogene, was upregulated in psoriasis lesions and linked to inflammatory skin diseases [46]. Additionally, several other pseudogenes and noncoding genes, including *RN7SL5P*, *CLCA3P*, and *LINC01698*, were identified, although their functions are not well understood. However, *CLCA3P* is part of the CLCA protein family, which regulates chloride channels and plays an important role in keratinocyte functions such as proliferation and apoptosis [47]. Finally, *C10orf67*, associated with sarcoidosis—an inflammatory condition affecting the skin through granuloma formation—was also identified [48]. These findings highlight distinct molecular mechanisms that may contribute to sex-specific differences in psoriasis pathogenesis.

Our analysis revealed that Tregs were elevated in psoriasis lesions, with a more pronounced increase in males. Tregs are known for their anti-inflammatory properties and critical role in maintaining immune homeostasis to prevent autoimmune diseases [49]. The overall rise in Tregs we identified in psoriatic lesions is consistent with previous studies, and it is hypothesized that their function may be impaired in psoriasis, leading to an increase in Th17 cells and contributing to disease pathogenesis. A correlation between Treg levels and disease severity has also been suggested, though the exact role of impaired Treg function in psoriasis remains unclear [50]. A recent study highlighted differences in expression of Treg markers between psoriasis and healthy samples [51]. We propose that Tregs may also exhibit distinct expression markers between males and females, potentially affecting disease pathogenesis.

Several studies have explored the role of DC subtypes in the development of psoriasis. cDCs can be classified into two types: CD1c+ DCs and CD141+ DCs. CD1c+ DCs are reduced in both non-lesional and lesional skin in psoriasis, while CD141+ DCs are increased in these areas [52,53]. In our analysis of lesional skin, we observed a greater reduction in cDCs in male psoriasis patients. Additionally, while iDCs are typically increased in psoriasis lesions, our data indicate a lower abundance of iDCs in males. These differences may indicate a sex-dependent effect on DC function.

Stromal cells, including fibroblasts and pericytes, were reduced in male psoriasis, indicating a potentially more severe disease progression due to impaired tissue repair and disrupted skin integrity. Neutrophils play a crucial role in psoriasis, serving as a histopathologic hallmark in lesions and contributing to disease development through processes such as respiratory burst, degranulation, and the formation of neutrophil extracellular traps (NETs) [54]. Cohen’s D analysis revealed a greater increase in neutrophils in male psoriasis patients compared to females. The abundance of neutrophils is particularly characteristic of severe forms such as generalized pustular psoriasis, and neutrophil depletion has shown efficacy in alleviating symptoms in pustular psoriasis in patients that did not respond well to conventional treatments [55]. Neutrophil processes like degranulation and NET formation are triggered via different stimuli, including cytosol calcium levels and ROS production, which can be influenced by sex hormones [56]. Additionally, pro-inflammatory M1 macrophages were also increased in males. Interestingly, in an estradiol knockout mouse model of psoriasis, treatment with estradiol, a major female sex hormone, reduced psoriatic inflammation by significantly decreasing the numbers of neutrophils and inflammatory macrophages [57], highlighting a potential anti-inflammatory role of estradiol in modulating these cell populations. Altogether, our finding that psoriatic lesions in males show a greater estimated increase in neutrophils and M1 macrophages is consistent with, and may potentially reflect, the increased severity of disease reported in male patients; however, this remains an observational association that requires validation through direct clinical measurements.

This study has several important limitations that should be carefully considered when interpreting the results. First, key clinical and demographic variables, including age, ethnicity, disease duration, PASI score, medication history, lifestyle factors, and biopsy location, were unavailable for most datasets. These factors are known to influence gene expression profiles, and their absence means that the observed transcriptomic differences between sexes cannot be attributed exclusively to biological sex. Some of the detected differences may reflect confounding by unmeasured clinical or demographic variables. Anatomical location and donor history of control biopsies were also unavailable, which may have introduced additional variability. It is therefore important to interpret all findings as sex-associated transcriptomic patterns rather than effects causally attributable to sex alone. Second, the 13 datasets integrated in this study were not originally designed to investigate sex-specific differences in psoriasis. This introduces substantial heterogeneity in experimental design, tissue handling, sequencing protocols, and patient characteristics. Although inter-study batch effects were corrected computationally (see Section 4.4), residual confounding from heterogeneous experimental conditions is likely unavoidable and represents an inherent limitation of this meta-analytic approach. Third, for nine of the 13 datasets, biological sex was inferred computationally rather than directly reported. Although MDS visualizations demonstrate consistent separation of inferred male and female samples (Supplementary Figure S1), the possibility of residual misclassification cannot be fully ruled out (see Section 4.3 for details). Fourth, this study is entirely observational and based on transcriptomic associations. No experimental or clinical validation is provided. All findings should therefore be regarded as hypothesis-generating rather than mechanistically conclusive. Future prospective, adequately powered cohort studies with complete clinical annotation and independent experimental validation will be required to confirm these findings.

Despite these limitations, the clear separation between control and lesional samples in MDS analyses and the consistency of identified pathways with well-established psoriasis biology support the overall validity of the findings as an exploratory analysis.

In summary, this exploratory, hypothesis-generating study provides transcriptomic evidence for sex-associated molecular and cellular differences in psoriasis pathogenesis. By identifying sex-associated DEGs, pathways, upstream regulators, and immune cell composition patterns, we highlight potential molecular distinctions between male and female psoriasis that may have relevance for precision medicine approaches. These patterns, however, are observational in nature and derived from transcriptomic associations alone. They should be regarded as candidate hypotheses rather than established mechanisms, and require confirmation through prospective studies with complete clinical annotation and independent experimental validation. Future research should prioritize sex-stratified analyses in larger, more diverse, and clinically well-characterized cohorts to determine the translational relevance of the patterns identified here.

## 4. Materials and Methods

### 4.1. Search Methods and Selection Criteria

A comprehensive search of the Gene Expression Omnibus (GEO) [58] and ArrayEpress (AE) [59] databases was conducted on 2 June 2024. The search terms included: ((“psoriasis”) OR (“psoriatic”)) AND (“high throughput sequencing” [Platform Technology Type]) AND “homo sapiens” [Organism] AND (“RNA-seq” OR “RNAseq” OR “RNA sequencing”). This systematic review was carried out in accordance with the PRISMA (Preferred Reporting Items for Systematic Reviews and Meta-Analyses) guidelines. A flowchart illustrating the study selection process is shown in Figure 1. Studies were included only if they met the following inclusion criteria: availability of RNA-seq raw data, transcriptomes derived from whole lesional or healthy control skin tissue samples (punch biopsy), sequencing conducted without additional manipulations (e.g., tissue culture, isolated cells), use of the Illumina sequencing platform, and availability of corresponding metadata. Studies utilizing microarray, single-cell RNA-seq (scRNA-seq), or small RNA-seq platforms were excluded.

Notably, control samples were obtained from normal, healthy individuals, including samples collected following cosmetic surgery, ensuring they represented non-psoriatic skin tissue. At the quality control stage, samples with fewer than 10 million mapped reads were excluded from downstream analysis (see Section 4.2). No additional sample-level exclusions were made beyond these pre-specified criteria.

### 4.2. RNA Sequencing Data Collection and Processing

RNA sequencing data from the selected studies were uniformly reprocessed. Raw data were downloaded using the SRA-Toolkit, version 3.0.1 (https://hpc.nih.gov/apps/sratoolkit.html (accessed on 2 June 2024)). In brief, the fasterq-dump tool was used to download FASTQ files, followed by adapter trimming and quality checks with Trim Galore, version 0.5.0 (https://www.bioinformatics.babraham.ac.uk/projects/trim_galore/ accessed on 15 September 2024) and FASTQC version 0.11.7 (https://www.bioinformatics.babraham.ac.uk/projects/fastqc/ accessed on 15 September 2024), respectively. High-quality reads were aligned to the reference genome (GRCh38) using HISAT2, version 2.1.0 [60], and the number of reads mapped to each annotated gene was counted with featureCounts, version 1.6.3 [61]. Only samples with a library size greater than 10 million counts were retained for statistical analysis, and genes with low read counts were filtered out using the ‘filterByExpr’ function in the edgeR package, version 3.38.1 [62].

### 4.3. RNA-Seq Sex Inference

To determine the biological sex of samples for which sex was not reported by the original authors, we used the SexInference GitHub repository (https://github.com/SexChrLab/SexInference.git accessed on 15 September 2024) to develop and test a logistic regression model with LASSO regularization for inferring biological sex from RNA-seq data. The model was implemented in R using the glmnet package [63,64] and incorporated the expression levels of six sex-chromosome genes as predictive features: *XIST* (X-linked, expressed exclusively in females as the master regulator of X-chromosome inactivation) and five Y-linked genes (*EIF1AY, KDM5D, UTY, DDX3Y*, and *RPS4Y1*), which are expressed exclusively in males. We trained and tested the model using gene expression data from the GTEx project (v8), which includes verified biological sex annotations. The optimal regularization parameter was selected by 10-fold cross-validation using cv.glmnet. Samples with predicted probability > 0.5 were classified as female; samples below this threshold were classified as male.

Model performance was assessed on a held-out 20% test partition of the GTEx data (n = 3475), yielding 100% accuracy, sensitivity, and specificity. To further validate the classifier on an external independent validation set, we identified all skin samples in our dataset for which biological sex had been directly reported by the original study authors. This yielded 46 samples across five studies: EMTAB6556, GSE47944, GSE67785, GSE183820, and GSE249936. The classifier was applied to these samples and predictions were compared against the author-reported sex labels. Results are summarized in Supplementary Table S1. Across all five studies, the model achieved 100% accuracy, sensitivity, and specificity (combined: 19 females and 27 males, zero misclassifications), providing strong independent validation of classifier reliability in skin tissue RNA-seq data. To further visualize the sex-specific expression structure of these 46 samples, we generated a heatmap of the six sex-inference genes following TMM normalization, voom transformation, and inter-study batch effect correction using removeBatchEffect. The heatmap clearly shows two distinct expression blocks corresponding to biological sex: *XIST* is highly expressed exclusively in female samples, while the five Y-linked genes are highly expressed exclusively in male samples, with near-zero expression in the opposite sex (Supplementary Figure S4). For the remaining samples in our dataset for which sex was not reported, the trained model was applied to infer biological sex, and MDS plots visualizing the normalized expression of six genes before and after the inference are depicted in Supplementary Figure S1. The inferred labels were used in all downstream analyses.

### 4.4. Differential Expression Analysis

Normalization and differential gene expression analysis were conducted using the edgeR, version 3.38.1 [65] and limma, version 3.52.1 [66] R packages. Briefly, raw read counts were normalized using the TMM method, followed by a voom transformation to approximate a normal distribution. This transformation produced a new dataset with values in logCPM (log2 counts per million), which were then used with classic normal-based statistical methods and workflows to detect differentially expressed genes (DEGs). Prior to initiating the differential expression analysis, we performed MDS analysis to visualize batch effects associated with the dataset in a reduced-dimensional space. We found that the “study source” factor made the largest contribution to the variation (Supplementary Figure S5). Consequently, the ‘study source’ variable was incorporated as a blocking factor in the limma design matrix, effectively modeling inter-study variation as a nuisance parameter during differential expression analysis. This approach is equivalent to including study as a covariate in a linear mixed model and is a standard method for batch effect correction in multi-study meta-analyses of transcriptomic data. Despite these corrections, we acknowledge that residual technical and biological confounding from the heterogeneous origins of these datasets is likely unavoidable and represents an inherent limitation of any multi-study integrative analysis. The dataset was divided into four groups: psoriasis male (PS_male), psoriasis female (PS_female), healthy control male (Control_male), and healthy control female (Control_female). DEG comparisons were performed separately for Psoriasis vs. Control male and Psoriasis vs. Control female. Additionally, we conducted a DEG analysis to study the interaction effect between sex and disease, represented by the contrast matrix interaction = (PS_male − Control_male) − (PS_female − Control_female). Statistically significant DEGs were defined as those with |log2 fold change (FC)| > 1 (either upregulated or downregulated) and an adjusted *p* value (Benjamini–Hochberg) < 0.05. Differences in gene expression distribution between groups were assessed using the Kruskal–Wallis test, followed by Dunn’s post hoc test.

### 4.5. Pathways Enrichment Analysis Using Metascape, IPA and WebGestalt

We performed pathway enrichment analysis using Metascape, IPA, and WebGestalt to explore biological processes and pathways relevant to psoriasis. First, we created Venn diagrams (https://bioinformatics.psb.ugent.be/webtools/Venn/ (accessed on 11 December 2024)) to compare the DEGs obtained from the two comparisons: control vs. psoriasis female and control vs. psoriasis male. This analysis allowed us to identify overlapping DEGs, as well as those unique to each comparison. We then used Metascape [67] (http://metascape.org/gp/index.html#/main/step1 (accessed on 11 December 2024)), a web-based tool integrating data from sources like Reactome, Canonical Pathways, BioCarta, GO Biological Processes, Hallmark Gene Sets, and KEGG Pathways, to perform enrichment analysis on the DEGs from each section of the Venn diagram. Metascape provided interactive visualizations and summary reports to highlight overrepresented biological terms and pathways. We displayed −log10(*p*-value) values greater than 2, corresponding to *p*-values less than 0.01, which are considered statistically significant. Additionally, we used IPA [68] (QIAGEN Inc., Redwood City, CA, USA; https://www.qiagenbioinformatics.com (accessed on 11 Decmber 2024)) to analyze upstream regulators associated with the DEGs from each comparison (males or females). IPA identified significant upstream regulators based on their ability to influence the expression of the DEGs. Negative z-scores indicated inhibition of regulators, while positive z-scores indicated activation. Regulators with *p*-values < 0.05 and absolute z-scores ≥ 2 were considered statistically significant. Finally, Gene Set Enrichment Analysis (GSEA) was conducted using WebGestalt (https://www.webgestalt.org (accessed on 11 December 2024) to identify pathways enriched in gender-specific psoriasis interactions. Genes were ranked based on differential expression analysis of the interaction term (PS_male − Control_male) − (PS_female − Control_female), highlighting pathways enriched or depleted in psoriasis males compared to females. Predefined gene sets were analyzed, and significant pathways were identified using FDR correction (adjusted *p* < 0.05). Results were visualized through barplots and enrichment plots.

### 4.6. Cell Type Enrichment Analysis Using xCell

xCell [69] (https://xcell.ucsf.edu/ (accessed on 26 November 2024)) is a web tool utilized for cell-type enrichment analysis, providing enrichment scores (ESs) for 64 immune and stromal cell types based on gene expression data. To perform the analysis, we first normalized the gene expression data using the Reads Per Kilobase per Million mapped reads (RPKM) method, which adjusts for gene length. We then addressed batch effects using the removeBatchEffect function. Our dataset was analyzed using xCell, including both control and psoriasis samples. A heatmap illustrating the average cell-type enrichment scores for each group (PS male, PS female, Control male, Control female) was generated using the ComplexHeatmap R package, version 2.12.1. Boxplots of the enrichment scores for each group were created and compared using Kruskal–Wallis analysis, followed by Dunn’s test for multiple pairwise comparisons, with a *p*-value < 0.05 considered statistically significant. Because xCell outputs relative enrichment scores rather than true cell proportions, these values were treated as ordinal measures of relative abundance. The Kruskal–Wallis test was therefore chosen as a non-parametric method appropriate for comparing distributions of enrichment scores without assuming proportionality of actual cell counts. To assess differences between male and female psoriasis, Cohen’s D scores were calculated to quantify the magnitude of these differences. Cohen’s D measures the effect size, indicating the extent of differences between the psoriasis and control groups for each sex. In this context, Cohen’s D reflects the magnitude of relative enrichment differences rather than absolute differences in cellular composition. For each comparison, the variances of Cohen’s D were estimated based on group sample sizes, and a z-statistic was computed to evaluate the difference in effect sizes between males and females for each cell type. *p*-values were derived from the z-statistics and corrected for multiple testing using the FDR, with FDR < 0.05 considered statistically significant. As xCell scores do not represent quantitative cell proportions, all statistical comparisons should be interpreted as contrasts in relative enrichment patterns rather than direct estimates of cellular abundance.

## Figures and Tables

**Table 1:** Study datasets by disease status and sex following sex determination.

No.	GEO\ArrayExpress Study	Psoriasis		Control	
		Female	Male	Female	Male
1	EMTAB6556	2	8	-	-
2	GSE117405 *	8	11	8	1
3	GSE121212 *	13	13	19	15
4	GSE171012 *	6	8	6	4
5	GSE176279 *	-	2	-	-
6	GSE183820	-	1	-	-
7	GSE205748 *	-	-	5	4
8	GSE249936	3	5	-	-
9	GSE41745 *	2	1	-	-
10	GSE47944	3	5	5	-
11	GSE54456 *	25	60	31	49
12	GSE67785	6	8	-	-
13	GSE83645 *	1	4	-	-
Total = 342		69	126	74	73

* Studies inferred sex via RNA-seq using a logistic regression model and six sex-specific genes.

**Table 2:** Upstream regulators unique to each sex in psoriasis vs. control. Red denotes activated upstream regulator; blue denotes inhibited upstream regulator.

Sex	Transcription Regulator	Ligand- Dependent Nuclear Receptor	Growth Factor	Transmembrane Receptor	Cytokine	Transporter	Ion Channel
Male	TBX21, STAT5A, REL, CREB1, KLF5, ACTN4, TOX, YAP1, YBX1, ATF6, C1QBPTBX2, STAT4, ETV4, MITF, ETS2, ELK1, VAV2, PRDM1, ATF3, RBL1, ARID1A, GATA6TRPS1, PPARGC1A, SATB1, MXD1, MYOCD	PGR, NR3C1	FGF2, VEGFA, NRG1, TGFB2	TNFRSF1B, Klrk1, IL2RG, IL11RA, ITGAV, ICAM1, CD80, PDCD1, LILRB1	TNFSF15, TNFSF14, CCL3, CXCL10, IFNE, CNTF	CEACAM1, TAP1, SLC6A4	CFTR
Female	STAT3, SP1, MSC, FOSL1, SOX2, POU2AF1, SOX11, GLIS1, COMMD3-BMI1, PAX1	AHR	FGF21	TNFRSF1A, TNFRSF10A, CHRNA1, LEPR		NPC1	KCNJ10

**Table 3:** Sex-specific DEGs in Psoriasis. Expression Status indicates changes in gene expression direction, based on comparisons between psoriasis and control samples, specific to each sex: statistically downregulated (Down) or upregulated (Up) in male (M) or in female (F) samples, or in both (F, M).

Gene Symbol	Gene Type	Location	Expression Status	Description & Role	Related Pathways
***SYT6***	Protein Coding	1p13.2	Down (M)	Calcium-dependent exocytosis of synaptic vesicles.	Calcium ion binding
***NELL2***	Protein Coding	12q12	Up (F)	Glycoprotein; prevents ER stress-mediated apoptosis.	Nervous system development, calcium ion binding
***PSG4***	Protein Coding	19q13	Up (M) Down (F)	Regulation of the innate immune system.	Platelet cytosolic Ca2+ response
***GPR15***	Protein Coding	3q11.2	Up (M, F)	Acts as a chemokine receptor.	GPCR signaling
***MAP1LC3C***	Protein Coding	1q43	Down (M, F)	Involved in selective autophagy.	Selective autophagy microtubule binding
***C10orf67***	Protein Coding	10p12.2	Down (M, F)	Chromosome 10 Open Reading Frame 67.	Data unavailable
***CLCA3P***	Pseudogene	1p22.3	Up (M, F)	Calcium-sensitive chloride conductance family.	Pseudogene
***CYP4Z2P***	Pseudogene	1p33	Up (M, F)	Includes heme and iron ion binding activity.	Pseudogene
***RN7SL5P***	Pseudogene	9p23	Up (F)	Non-coding RNA; 7SL cytoplasmic component.	Pseudogene
***LINC01698***	lncRNA	1q32.2	Down (M, F)	Long intergenic non-protein coding RNA.	Regulatory functions

## Data Availability

The original contributions presented in this study are included in the article/Supplementary Material. Further inquiries can be directed to the corresponding author.

## Supplementary Materials

The following supporting information can be downloaded at: https://www.mdpi.com/article/10.3390/ijms27104439/s1.
