# Supplementary material for: Sex-Specific Transcriptomic Profiles in Psoriatic Lesions: A Large-Scale Integrative Study
Source: Int J Mol Sci. 2026 May 15;27(10):4439. doi: 10.3390/ijms27104439 (PMC13207142; doi:10.3390/ijms27104439)
Supplement: Supplementary file 1 [file ijms-27-04439-s001.zip › ijms-4245573-supplementary.pdf]

Supplementary Materials

Sex Specific Transcriptomic Profiles in Psoriatic Lesions: A Large-Scale Integrative Study

*Corresponding author: Mali Salmon-Divon, Department of Molecular Biology, Ariel University, Kiryat Hamada 3 Ariel, Israel 40700. email: malisa@ariel.ac.il.

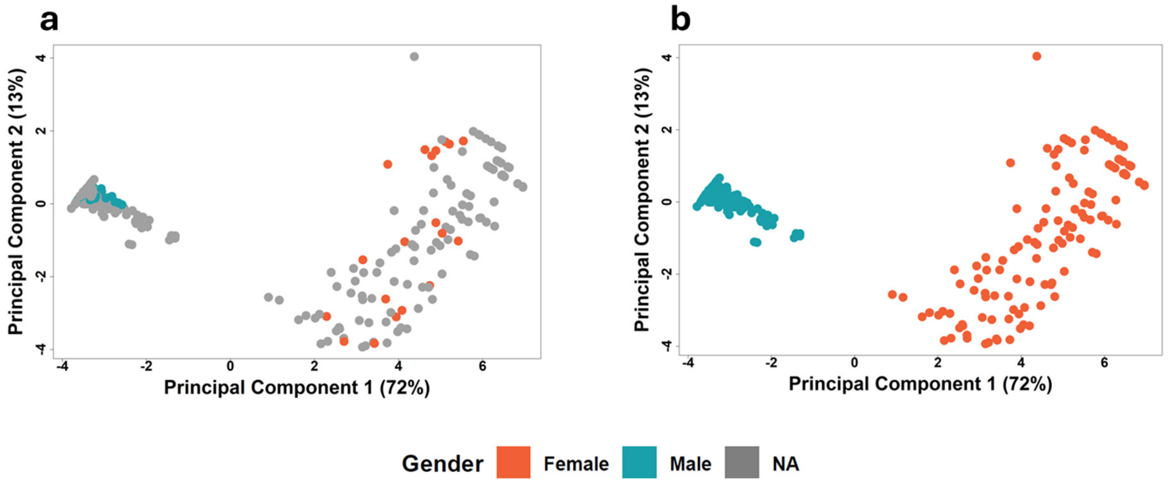

Supplementary Figure S1: The Multidimensional Scaling (MDS) plot visualizes the normalized expression of six genes: the female-specific gene XIST and the Y-linked genes EIF1AY, KDM5D, UTY, DDX3Y, and RPS4Y1, to determine sample sex. (a) Before inferred sex (b) After sex inference. Colors represent Male (Blue), Female (Orange), and NA (Grey).

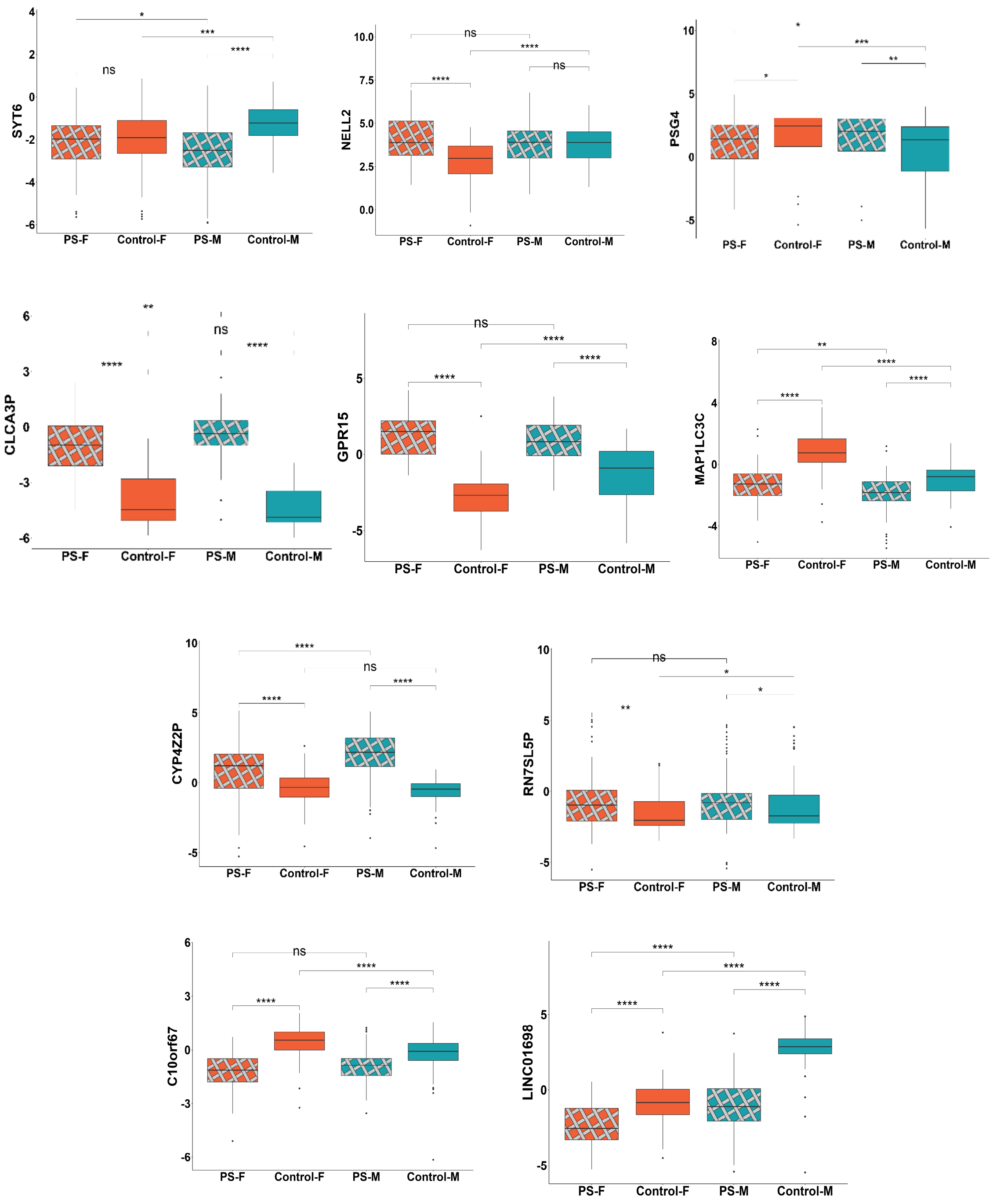

<u>Supplementary Figure S2:</u> Boxplot of normalized gene expression after study batch removal, showing the distribution of 10 interaction genes across all cohort groups: Psoriasis female (PS-F), Control female (Control-F), Psoriasis male (PS-M), and Control male (Control-M). Kruskal-Wallis test followed by Dunn's test was used to calculate statistics. Asterisks indicate significance: *p < 0.05, **p < 0.01, ***p < 0.001, ****p < 0.0001, and "ns" for non-significant.

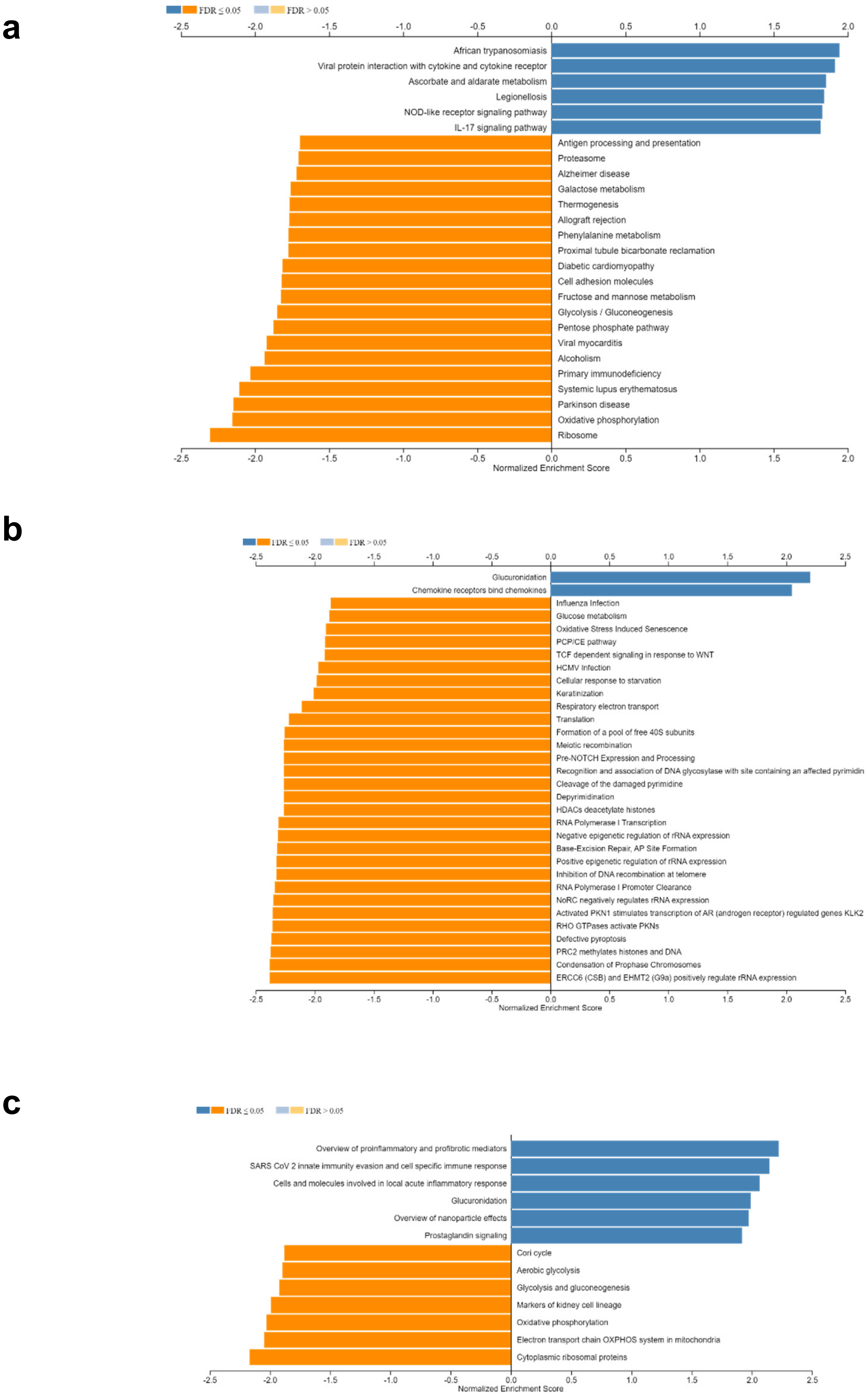

<u>Supplementary Figure S3:</u> Barplot showing pathways significantly enriched or depleted in psoriasis males compared to females. Genes were ranked based on the interaction term (PSmale−Controlmale)−(PSfemale−Controlfemale)(PS_male - Control_male) - (PS_female - Control_female)(PSmale−Control_male)−(PS_female−Control_female), and pathways were identified using GSEA via WebGestalt. Significance was determined using FDR correction (adjusted $p < 0.05$). Bars represent the -log10(FDR-adjusted p-value) of each pathway.Pathway analysis of interaction DEGs using WebGestalt (https://www.webgestalt.org, PMID: 38808672). Results are shown for the KEGG database (a), Reactome database (b), and WikiPathways (c).

Supplementary Table S1: Performance of the sex-inference classifier on GTEx training data and on independent skin datasets with author-reported biological sex.

Dataset	Tissue	N	Female	Male	Accuracy (%)	Sensitivity (%)	Specificity (%)
GTEx (10-fold CV)	Various	17,382	5,798	11,584	100.0	100.0	100.0
Combined (EMTAB6556, GSE47944, GSE67785, GSE183820, GSE249936	Skin	46	19	27	100.0	100.0	100.0

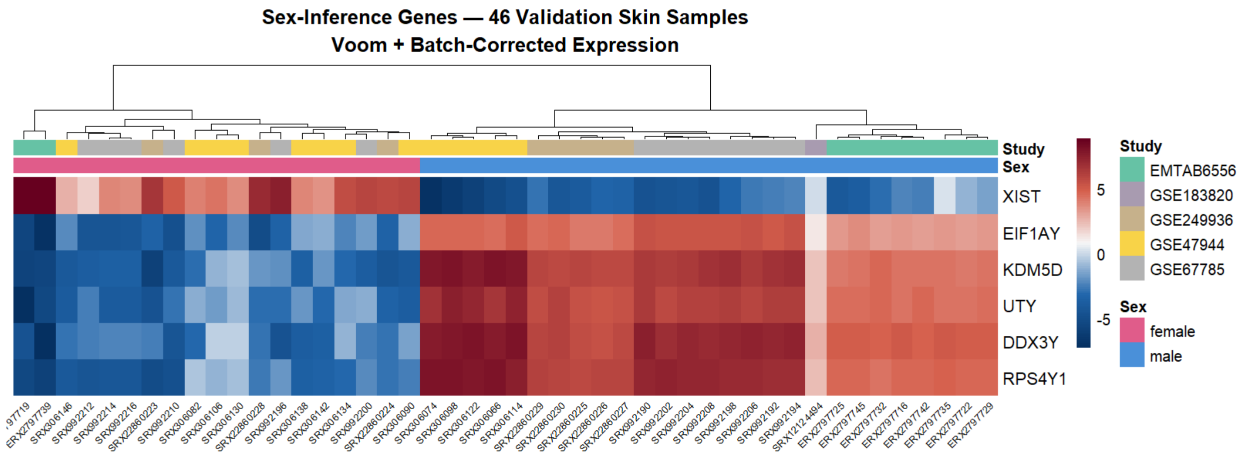

Supplementary Figure S4: Heatmap of sex-inference gene expression across 46 validation skin samples with author-reported biological sex. Rows represent six sex-chromosome genes (XIST and five Y-linked genes: EIF1AY, KDM5D, UTY, DDX3Y, RPS4Y1). Columns are samples clustered unsupervised by Euclidean distance. Expression values are voom-transformed, TMM-normalised, and batch-corrected. Colour scale: blue = low, red = high expression. Annotation bars indicate reported sex and study. Unsupervised clustering naturally separates female and male samples into two distinct groups based on their mutually exclusive expression of X- and Y-linked markers.

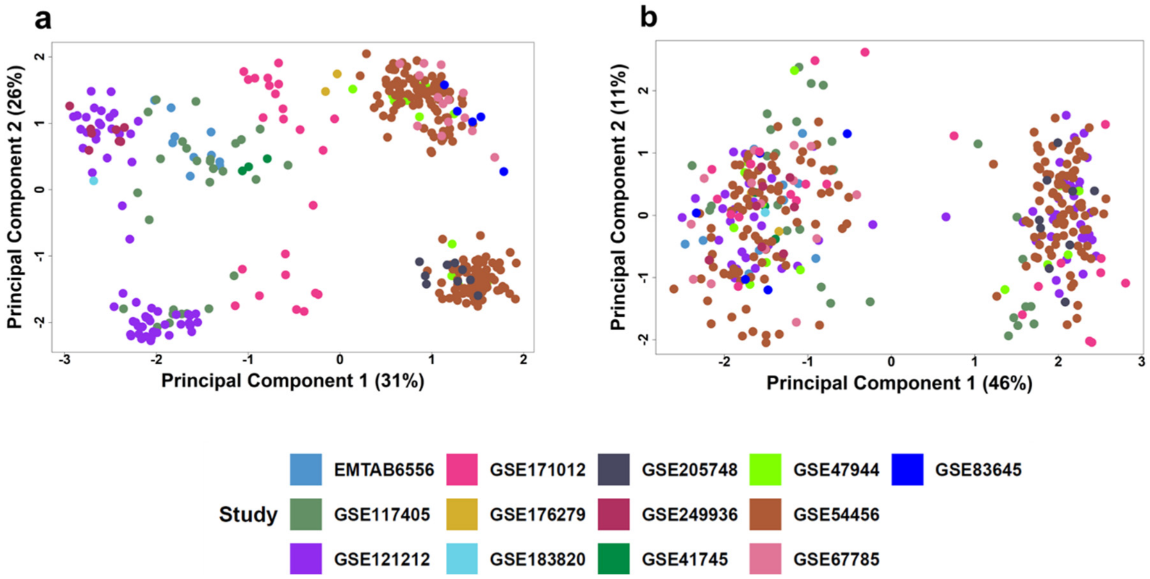

Supplementary Figure S5: The Multidimensional Scaling (MDS) plot visualizes the normalized expression of the samples before study batch (a) and after remove study effect(b)
